# The Benefits of Physical Activity in Individuals with Cardiovascular Risk Factors: A Longitudinal Investigation Using fNIRS and Dual-Task Walking

**DOI:** 10.3390/jcm10040579

**Published:** 2021-02-04

**Authors:** Deborah Talamonti, Thomas Vincent, Sarah Fraser, Anil Nigam, Frédéric Lesage, Louis Bherer

**Affiliations:** 1 Montreal Heart Institute, Research Centre and Centre EPIC, Montreal, QC H1T 1N6, Canada; thomas.tv.vincent@gmail.com (T.V.); anil.nigam@icm-mhi.org (A.N.); frederic.lesage@polymtl.ca (F.L.); louis.bherer@umontreal.ca (L.B.); 2 Interdisciplinary School of Health Sciences, University of Ottawa, Ottawa, ON K1N 6N5, Canada; Sarah.Fraser@uottawa.ca; 3 Department of Medicine, Université de Montreal, Montreal, QC H3T 1J4, Canada; 4 École Polytechnique de Montreal, Montreal, QC H3T 1J4, Canada; 5 Centre de Recherche, Institut Universitaire de Gériatrie de Montréal, Montréal, QC H3W 1W5, Canada

**Keywords:** fNIRS, cardiovascular, aging, dual-task, gait speed, walk, executive functions

## Abstract

Cardiovascular fitness is linked to better executive functions, preserved gait speed, and efficient cortical activity. Older adults with cardiovascular risk factors (CVRFs) typically show poor cognitive performance, low physical fitness, and altered brain functioning compared with healthy individuals. In the current study, the impact of regular physical activity on cognition, locomotion, and brain functions was explored in a cohort of older adults with low or high CVRFs. Cortical activation of the frontal areas was investigated using functional Near-Infrared Spectroscopy (fNIRS) at baseline, at 6 months and at 12 months. Evoked cortical response and behavioral performance were assessed using the dual-task walking paradigm, consisting of three conditions: single cognitive task (2-back task), single walking task (walking), and dual-task (2-back whilst walking). Results show greater task-related cortical response at baseline in individuals with high CVRFs compared to those with low CVRFs. Moreover, participants with high CVRFs benefitted the most from participating in regular physical activity, as their cortical response decreased at the 12-month follow-up and became comparable to that of participants with low CVRFs. These changes were observed in conjunction with improved cognitive performance and stable gait speed throughout the 12-month period in both groups. Our findings provide evidence that participation in regular physical activity may be especially beneficial in individuals with CVRFs by promoting brain and cognitive health, thus potentially contributing to prevention of cognitive decline. Future research may explore whether such effects are maintained in the long-term in order to design ad-hoc interventions in this specific population.

## 1. Introduction

The aging process causes deleterious changes in the brain that are related to a decline in several cognitive and physical domains, and may culminate in cognitive decline and dementia [1]. These changes are exacerbated in individuals with Cardiovascular Risk Factors (CVRFs), such as hypertension, type 2 diabetes, smoking, and obesity [2]. Multiple CVRFs have been linked to worse cognitive performance on tests of executive functions, processing speed, and verbal memory in middle age [3] and older adulthood [4]. For instance, in a population-based cohort study of over 8000 older adults, Dregan et al. [4] observed that individuals with more severe CVRFs had significantly lower global cognition, executive functions, and memory after 4-year follow-up compared to participants with less severe CVRFs. CVRFs have also been linked to increased risk of cognitive impairment and dementia [5,6,7,8].

Recent neuroimaging studies have observed that CVRFs are related to structural and functional brain alterations, mostly of the fronto-parietal and temporal areas, such as reduced grey matter volume [9], and altered activity of the fronto-parietal lobes [10]. CVRFs have also been suggested to accelerate structural brain changes in older adults, causing infarcts or atrophy [9,11] that may be linked to neurodegeneration in this population [3]. Finally, CVRFs cause alterations of the normal cerebral blood flow [12,13]. For instance, using functional Near Infrared Spectroscopy (fNIRS) during dual-task walking, Holtzer et al. [14] recently observed increased prefrontal activation, but lower behavioral performance, in older adults with type 2 diabetes compared to healthy peers. Similarly, greater prefrontal activation, but equivalent performance was reported during a precision walking task in obese individuals [15]. This overactivation has been suggested to be related to decreased brain resources as a consequence of the neuropathy in patients with CVRFs. Both type 2 diabetes and hypertension have been associated with decreased walking speed [16], showing that the general burden of CVRFs may reflect not only on cognition and brain functioning, but also on mobility.

In the general population and in individuals with cardiovascular diseases, physical activity has been proven to increase both cognition and mobility, to prevent future cognitive decline, and to improve cortical response in the brain [2,17,18,19,20]. Increased evidence shows that regular physical activity in older adults reduces the severity of CVRFs, such as diabetes [21], obesity [22], and hypertension [23,24], and increases cardiovascular fitness. However, despite the clear evidence supporting the benefits of physical activity on CVRFs, many questions regarding the effects of physical activity on cognition, mobility, and brain functionality remain unanswered [25]. Given the benefits reported in other populations, it is plausible that physical activity in older adults with CVRFs may improve cognition and mobility, and may reduce the brain overactivation reported in previous studies on single CVRFs [14,15]. Moreover, although CVRFs often co-exist [26] and may affect brain functioning cumulatively, no research has so far investigated the burden of multiple CVRFs on the brain.

In the current study, the benefits of regular physical activity on the brain were explored in a population of older adults with either low or high CVRFs during a period of 12 months, by means of fNIRS. Moreover, the effects of regular physical activity on cognition and mobility (i.e., gait speed) were explored using the dual-task walking paradigm, a task of executive functions that compares performance at a cognitive task alone, a walking task alone, and simultaneous cognitive and walking tasks. This test is particularly suited to investigate subtle changes in walking mechanisms and performance at tests of executive functions, both predictors of cognitive decline [27] and linked to age-related brain changes in healthy older individuals [28]. Our results indicated that individuals with high CVRFs showed slower walking speed and increased task-related cortical response at baseline. However, participating in regular physical activity had a positive effect on behavioral outcomes and brain hemodynamics, specifically in those with high CVRFs, as they showed reduced cortical activation at follow-up visits, indicating greater brain health, whilst improving their cognitive performance at follow-up visits.

## 2. Experimental Section

Participants were registered members of the preventive medicine and physical activity center (Centre EPIC) of the Montreal Heart Institute, one of the largest cardiovascular prevention centers in Canada and in North America. Participants for this study were selected if aged 60 or above, with normal or corrected vision, normal hearing, and able to walk 15 m without assistance. Exclusion criteria were as follows: alcohol consumption >2 drinks per day, abnormal cognitive functioning (Mini-Mental State Examination; MMSE < 26), history of neurological, cardiac, chronic or psychiatric diseases, major surgery within one year of enrollment, non-cardiopulmonary limitation to exercise, corrected or uncorrected hearing problems, and use of psychotropic medication known to affect cognition. The twenty-four selected participants were divided into two groups: low CVRFs (LCVRF) and high CVRFs (HCVRF). Groups were classified based on the Framingham score [29], where variables such as age, sex, cholesterol, blood pressure, diabetes mellitus, and smoking status are utilized to calculate 10-year risk of developing heart diseases. Individuals with HCVRF had low to moderate risk in at least two of the considered indexes: high cholesterol, diabetes, hypertension, low high-density lipoprotein, high low-density lipoprotein, and smoking. Individuals identified as LCVRF had none or very low risk in only one of the above indexes. 

At baseline (T0), participants signed the informed consent for participation in the study and completed clinical, neuropsychological, and physical assessments, followed by the dual-task walking paradigm, where the cortical response was recorded by means of a portable fNIRS system. Following this, participants were asked to freely take part in any exercise training prescribed by registered kinesiologists as part of their preventive programs at the ÉPIC center. Following the international recommendations for exercise testing and prescription [30], participants were asked to take part in regular physical activity at the ÉPIC center at least twice per week for a duration of 12-month. Measures of weekly frequency, duration, and intensity of physical activity were obtained through self-report questionnaires. For the intensity of the physical activity, the Borg scale of perceived exertion was used [31]. After 6 months, a first follow-up visit was scheduled for the neuropsychological assessment, including the dual-task walking paradigm and fNIRS recording (T6). These assessments were also repeated at 12-month follow-up (T12). These follow-ups were chosen to better document changes in cognition and the impact of PA overtime [32,33]. This study was approved by the Montreal Heart Institute ethic committee (ICM#12-1386), and completed in accordance with the Helsinki Declaration. 

The dual-task paradigm consisted of three experimental conditions: single cognitive task (SC), single walking task (SW), and dual-task (DT). SC required participants to perform the 2-back working memory task while standing. Participants listened to a series of numbers at a constant presentation rate of 1.5 s and were asked to recall the number they heard two positions back. During SW, participants were asked to freely walk back and forth on a 10-m track for 30 s. During DT participants were asked to complete the auditory 2-back task whilst walking. Auditory cues were presented through a headset. Each experimental condition was administrated in blocks of 30 s, with periods of fNIRS baseline lasting 5 s before each block and 15 s after each block. The complete sequence of stimulation blocks was presented following an ABBA design, as follows: SC–SC–SW–DT–DT–DT–DT–SW–SC–SC. This type of design was chosen in order to control for fatigue effects across the conditions. Mean values of accuracy for the 2-back (% correct) and walking speed (*m*/*s*) from the cognitive and motor blocks were calculated, resulting in two variables (cognitive performance and gait speed) and two conditions (single and dual-task).

**Figure 1:**
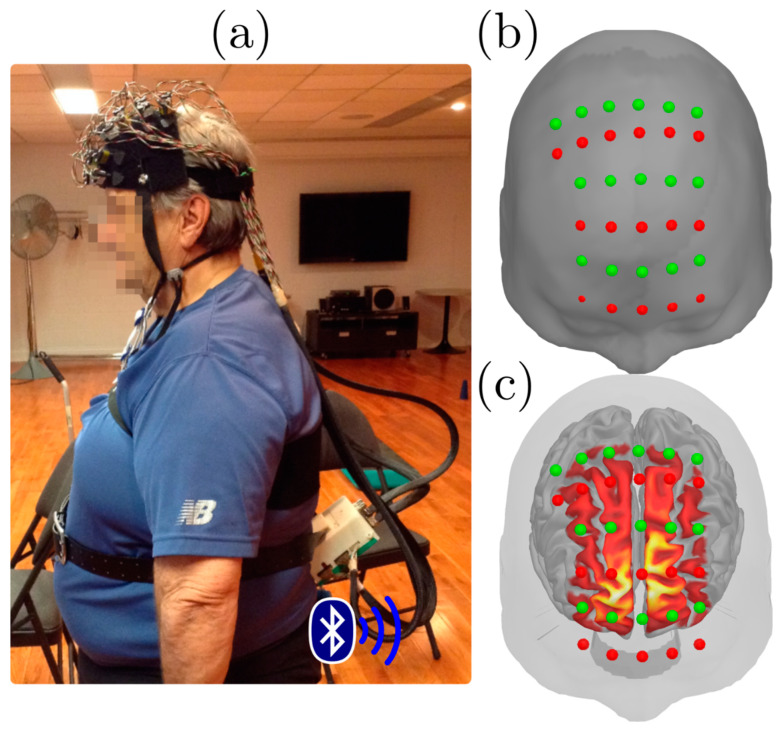
Functional Near-Infrared Spectroscopy (fNIRS) experimental setup. (**a**) Participant wearing the NIRS device consisting of a frontal cap wired to a Bluetooth acquisition device (**b**) Frontal view of the optode layout with sources in red and detectors in green, overlaid on the Colin27 anatomical template. (**c**): cortical sensitivity map for the fNIRS measurements covering the central part of the prefrontal cortex, overlayed with fNIRS montage and the scalp surface.

During the dual-task experiment, fNIRS light intensity signals (wavelengths 735 nm and 860 nm) were recorded at a sampling rate of 20 Hz using an fNIRS portable system whose sensors covered the front of the head. This device was built by a team at École Polytechnique in Montreal, Canada [34]. It consisted of LED sources and Avalanche PhotoDetectors attached to a frontal cap and connected to an acquisition device positioned on the participant’s belt. Data were streamed via Bluetooth to software developed in LabVIEW (National Instruments). The montage comprised 16 sources and 16 detectors coupled into 128 pairs (256 channels), with separations ranging from 2.5 to 3 cm. The optode layout was designed to maximally cover the prefrontal cortex (PFC) including partial coverage of pre-motor areas. Figure 1 depicts the acquisition setup, as well as the optode layout and cortical coverage.

**Figure 2:**
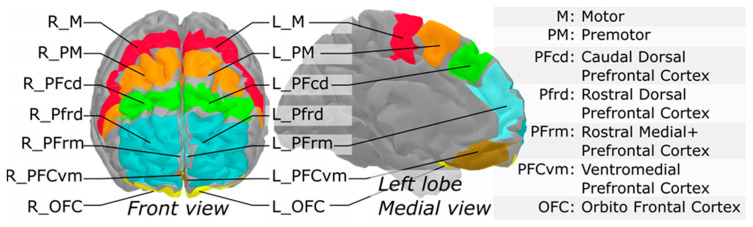
Segmentation of the prefrontal cortex based on MarsAtlas, used to produce region-averages of NIRS task-related effects.

fNIRS data were processed using brainstorm [35] and the nirstorm plugin [36], under Matlab 2017. Pre-processing steps were performed in the channel-space and first involved motion correction using spline interpolation within manually tagged periods of motion artefacts [37]. The following additional steps were applied: automatic removal of glitches due to occasional short-lived signal interruptions, as well as bandpass filtering between 0.01 and 0.1Hz to remove fluctuations unrelated to evoked hemodynamic events. Channel time-series were then projected on the cortical surface of the Colin27 template [38] using the Minimum Norm Estimate algorithm [39]. A first-level GLM with a pre-colored noise model [40] was applied to the cortical time-series of each subject to obtain within-subject t-stat mappings of (HbO) (oxygenated hemoglobin) and (HbR) (deoxygenated hemoglobin) changes evoked by the three experimental tasks (SW, SC and DT). Since the NIRS spatial resolution is relatively low, mesh-based cortical mappings contain a lot of redundant information. To get a more parsimonious representation of NIRS mappings, regional averages were computed using a coarse version of the MarsAtlas cortical parcellation [41]. This segmentation consisted of a set of 14 regions (7 per hemisphere), as depicted in Figure 2 with the list of region labels. Lastly, to keep only the areas that were potentially engaged in the experimental paradigm, task-specific functional masks were computed from a group-level analysis. To do so, a second-level GLM with a mixed-effect noise model [40] was applied to produce binary maps from t-stats thresholded at *p* < 0.05 (uncorrected). For each experimental condition, this allowed filtering out the regions which elicited no activity at the group-level. At the end of the NIRS processing pipeline, the within-subject and region-specific t-values were used as task-related hemodynamic responses to investigate their relationship with the other study variables in the following statistical analyses.

A series of t-tests and two mixed 2 (group: LCVRF, HCVRF) × 3 (time: T0, T6, T12) × 2 (condition: single task, dual-task) ANOVAs were performed to explore between-group differences and the effect of time and condition on cognitive performance and gait speed respectively. A series of two (group: LCVRF, HCVRF) × 3 (time: T0, T6, T12) ANCOVAs were performed on each activated region, averaged over hemisphere, to investigate whether the presence of high CVRFs and participation in regular physical activity impacted cerebral hemodynamics during SC, SW and DT, whilst controlling for sex. All assumptions to run the performed analyses were met, unless otherwise stated. Post-hoc pairwise comparisons were performed using Bonferroni correction.

## 3. Results

Table 1 shows demographic, clinical characteristics, and groups comparisons of participants at baseline. Data are mean ± standard deviation for continuous variables and count for categorial variables. Those classified as the LCVRF group were 14 (60.87%), whereas nine had HCVRF (39.13%). Of the total sample, 15 (65.2%) were women. Mean age at baseline was 68.00 ± 5.78 years and mean level of years of schooling was 15.52 (± 4.24). All participants were cognitively well functioning (MMSE = 28.39 ± 3.70), and had low scores on the geriatric depression scale (GDS), indicating no major depressive symptoms (GDS = 4.61 ± 3.70). There were no statistically significant differences between the two groups for the demographic variables. Moreover, the self-reported perception of completed physical activity (i.e., frequency, duration, and intensity) did not show significant differences between the two groups throughout the duration of the study.

### 3.1. Behavioral Results

Two 2 (condition: single-task, dual-task) × 2 (group: LCVRF, HCVRF) × 3 (time: T0, T6, T12) ANOVAs were performed to investigate between-group differences and the effect of time and condition on cognitive performance and gait speed. Data are mean ± standard deviation, unless otherwise stated.

**Figure 3:**
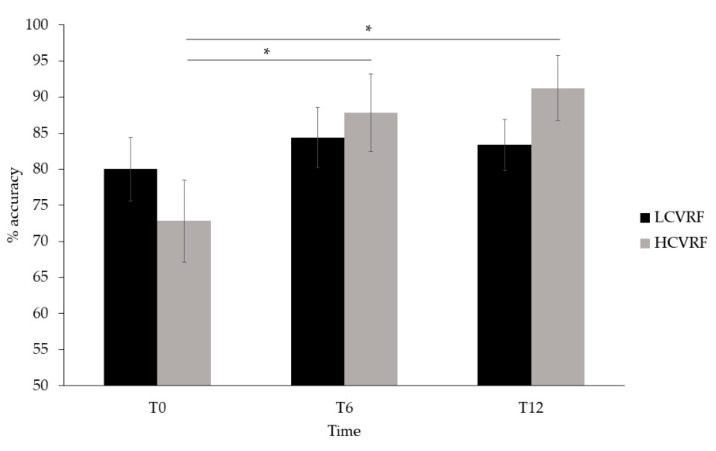
General cognitive performance in low-risk and high-risk cardiovascular risk factors (LCVRF, HCVRF) at baseline (T0), 6 months (T6), and 12 months (T12) follow-ups. Bars indicate standard error. * = *p* < 0.05.

Regarding cognitive performance, two outliers were found in the data, as assessed by inspection of a boxplot, whose performance was considerably lower (15% accuracy) than the average (83%). The outliers were removed from the analysis because they materially affected the results, as assessed by a comparison of the results with and without the outliers. Specifically, the analysis with the outliers revealed no statistically significant results (*p* values > 0.05). The analysis without the two outliers revealed a significant main effect of time (Greenhouse–Geisser corrected: F(2, 38) = 11.913, *p* < 0.001 η^2^_p_ = 0.385), in which cognitive performance was greater at T6 (86.12 ± 3.40) and T12 (87.31 ± 2.90) than at T0 (76.41 ± 3.60) (T6: *p* = 0.001; T12: *p* = 0.002). No main effects of group or condition were found. The main effect of time was qualified by an interaction between time and group (F(1, 38) = 4.986, *p* = 0.012, η^2^_p_ = 0.208), in which the HCVRF group performed significantly better at T6 (87.81 ± 5.35) and at T12 (91.25 ± 4.56) than at T0 (72.81 ± 5.67) (both *p* = 0.001), as shown in Figure 3. Such difference was not found in LCVRF, who remained stable throughout the study. In addition, a significant time by condition interaction (Greenhouse–Geisser corrected: F(2, 38) = 6.269, *p* = 0.010, η^2^_p_ = 0.248) demonstrated that cognitive performance was greater at T6 (88.57 ± 3.60) and at T12 (85.71 ± 3.39) than at T0 (72.80 ± 4.40) during SC (T6: *p* = 0.001; T12: *p* = 0.019), and at T12 (88.91 ± 2.80) compared to T0 (80.01 ± 3.54) during DT (*p* = 0.007). No other significant interactions were found. 

**Figure 4:**
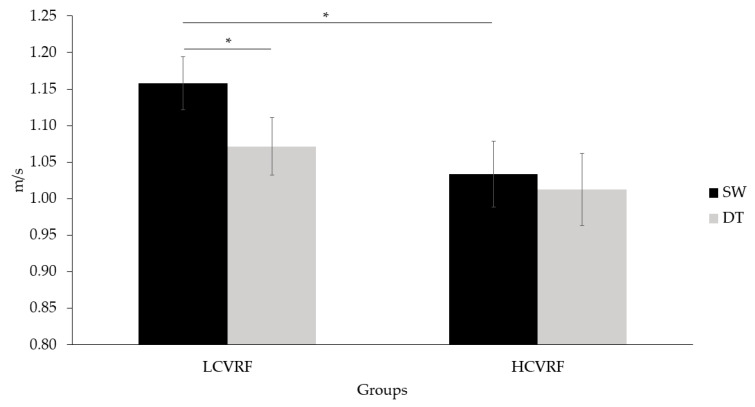
Gait speed at single walking (SW) and dual-task (DT) conditions in participants with low (L) and high (H) cardiovascular risk factors (CVRF) averaged across all time points. Bars indicate standard error. * = *p* < 0.05.

Regarding gait speed, a main effect of condition was observed (F(1, 20) = 17.795, *p* < 0.001, η^2^_p_ = 0.459), in which both groups walked faster during SW (1.096 ± 0.029) compared to DT (1.042 ± 0.032) (*p* < 0.001). There were no main effects of group or time. A significant group by condition interaction was found (F(1, 21) = 6.545, *p* = 0.018, η^2^_p_ = 0.238). The contrast between conditions revealed that the LCVRF group walked faster during SW (1.158 ± 0.036) than during DT (1.072 ± 0.040) (*p* < 0.001), whereas the group with HCVRF did not show such a pattern. The contrast between groups revealed that the LCVRF group walked faster (1.158 ± 0.036) than the HCVRF group (1.034 ± 0.045) during SW (*p* = 0.044) (see Figure 4). No other significant interactions were found.

### 3.2. fNIRS Main Effect and Functional Mask

**Figure 5:**
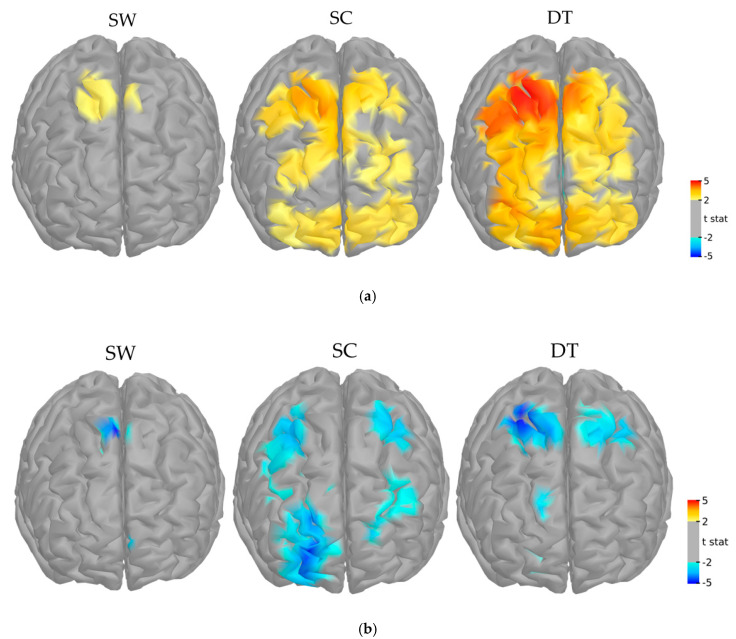
Group-level cortical mappings of HbO (**a**) and HbR (**b**) changes evoked by single walking (SW), single cognitive (SC) and dual-task (DT) conditions computed from all subjects and time points, threhsolded at *p* < 0.05 (−0.5 to +0.5). These mappings were used as functional masks to extract within-subject region-specific effects.

The main task-related fNIRS effects for both groups and all time points showed a bilateral activation of frontal areas that was proportionate to the complexity of the tasks (Figure 5). For HbO responses, cortical activation was lower during SW, limited mostly to the premotor and motor areas (PM, M, PFcd), and greater during SC and DT, where both prefrontal and motor regions were recruited (PFrm, PFcd, PFrd, PM, M). For HbR responses the hemodynamic response was limited to the premotor area during SW and to motor and prefrontal regions during SC and DT (PFcd, PFrd, PM, M).

### 3.3. Interaction between fNIRS, Clinical Group and Time

**Figure 6:**
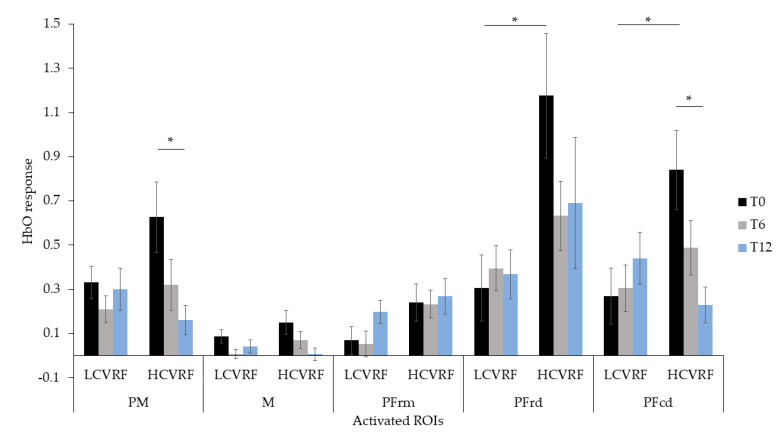
Cortical activation during the SC (Single Cognitive) condition in participants with low (L) and high (H) cardiovascular risk factors (CVRF). Values shown in Δμmol/L, ranging between −0.1 to +1.5. Bars indicate standard error. * = *p* < 0.05.

HbO responses at each time point and condition and in each group are presented in Table 2. Regarding the SC condition, the ANCOVAs showed a main effect of time (T0 > T12) in PM (F(2, 40) = 6.253, *p* = 0.004, η^2^_p_ = 0.238) and in M (F(2, 40) = 4.015, *p* = 0.026, η^2^_p_ = 0.167). A main effect of group (HCVRF > LCVRF) was observed in PFrd (F(1, 20) = 5.771, *p* = 0.026, η^2^_p_ = 0.224). Significant Time × Group interactions were found in the PM (F(2, 40) = 3.977, *p* = 0.027, η^2^_p_ = 0.166) and the PFcd (F(2, 40) = 5.578, *p* = 0.007, η^2^_p_ = 0.218). Post-hoc comparisons revealed that the HCVRF group showed greater cortical activation than the LCVRF group in the PFcd at T0 (F(1, 20) = 6.592, *p* = 0.018, η^2^_p_ = 0.248). The HCVRF group also showed greater response at T0 than at T12 in both PM (F(2, 19) = 8.472, *p* = 0.002, η^2^_p_ = 0.471) and PFcd (F(2, 19) = 5.319, *p* = 0.015, η^2^_p_ = 0.359). The change from T0 to T12 was not observed in the LCVRF group. These results are shown in Figure 6 and suggest that, at baseline, participants with high CVRFs showed more activation in the premotor and prefrontal regions when performing the single n-back. Such overactivation also decreased after 12 months of participation in regular physical activity.

No significant effects of time, group, or time by group interactions were observed in HbO responses during SW.

Regarding the DT condition, the ANCOVAs showed a main effect of time (T0 > T6 and T12) in the PM (F(2, 40) = 4.024, *p* = 0.026, η^2^_p_ = 0.168) and an effect of group (HCVRF > LCVRF) in the PFrd (F(1, 20) = 5.771, *p* = 0.026, η^2^_p_ = 0.224). There were no additional significant main effects or interactions.

Finally, for HbR responses there were no significant main effects of time or group and no significant time by group interactions.

## 4. Discussion

The present study investigated changes in behavioral and cortical activation patterns over a period of 1 year in individuals with low and high CVRFs taking part in regular physical activity. Behavioral results showed improvements in cognitive performance throughout the study in both groups, thus suggesting the positive impact of physical activity, which all participants practiced regularly, above the 150 min/week recommendation [42], for the full duration of the study. On a cortical level, all participants, but especially those with HCVRF, demonstrated a more efficient hemodynamic response during the 1-year period of participation in regular physical activity, as a decrease of task-related cortical activation was observed at follow-up visits, with improvement of cognitive performance.

The improvements in cognitive performance observed at follow-up visits reinforce the importance of physical activity in older adulthood as a potential protector against cognitive decline. The improvement was more evident in HCVRF, suggesting cognitive benefits in this specific group. These results are in line with previous investigations in older adults at risk of dementia and cardiovascular diseases [43,44]. For instance, Espeland et al. [44] explored the effect of a 24-month physical activity intervention on physical and cognitive functions in older adults with and without diabetes. They found that at 24-month follow-up, diabetic participants assigned to the physical activity intervention performed better at tests of various cognitive domains, including executive functions. Our study extended these findings to a population with multiple CVRFs and by including a measure of task-related cortical activation.

Participants with HCVRF also walked slower than those with LCVRF during the dual-task paradigm and did not show any difference in gait speed between the single and dual component of the dual-task, as typically observed in healthy individuals [45] and as shown by the LCVRF group. The absence of differences in gait speed between single and dual-tasks and the slow gait speed shown by those with HCVRF are in line with previous studies [16] and suggest the clinical value of gait disturbances in individuals with CVRFs. Those with LCVRF showed decreased gait speed during DT than SW. This pattern suggests that the cognitive component of the dual-task is prioritized over the walking component, in line with the existing literature (for a review, see [43]). The behavioral findings are of clinical relevance because they show that participation in regular physical activity has the potential to delay the appearance of cognitive impairment in individuals at high risk of developing dementia.

fNIRS data showed task-related hemodynamic response [46,47,48]. Cortical activation was lower during SW, greater during SC, and increased significantly during DT in all participants and at all time points. This pattern is in line with previous studies exploring the neural bases of dual-task experimental designs (for a review, see [46]). Bilateral rostral and caudal dorsal areas of the prefrontal cortex were especially involved during SC, whereas premotor and supplementary motor areas were activated during SW. Based on the MarsAtlas [41] cortical parcellation utilized in the current study, those regions corresponded approximately to Brodmann areas (BA) 8, 9, 10, and 46 [49]. BA 8 is typically involved in the execution of complex motor and cognitive tasks, including initiating, coordinating, and planning sequences of movements [50], whereas BA 9, 10, and 46 are more involved in memory processes, such as encoding, retrieval, and working memory [51]. It is thus not surprising that these areas were activated during the SC and the DT components of the dual-task, which are tasks of working memory and a clear example of multi-tasking.

During SW, regions related to mobility processes were activated in all participants and included the motor, premotor, and dorsal caudal prefrontal cortices. No effects of time or group were found on cortical activation during this task. Although the frontal areas are related to walking mechanisms in younger adults, a loss of automaticity in older adults has been shown to cause a greater recruitment of posterior areas as a compensatory strategy [52]. It is thus possible that the cortical activity related to SW in our sample may have been mediated by areas outside of the investigated ROIs. Future research may consider alternative channel configurations in order to explore a wider area of the neocortex in dual-task designs.

The hemodynamic response during SC and DT was greater in HCVRF than in LCVRF at baseline specifically in prefrontal caudal and rostral dorsal regions. These results are in line with existing investigations showing brain imaging changes in individuals at greater risk of cardiovascular diseases (for a review, see [12]). For instance, using fMRI during a test of executive functions, Chuang et al. [10] observed greater task-related parietal activation in individuals with higher CVRF. Similarly, Holtzer et al. [14] observed a greater hemodynamic response during the SC component of a dual-task walking paradigm in diabetic patients. In our study, a greater hemodynamic response in participants with HCVRF was related to equivalent cognitive performance to that of the LCVRF group, thus suggesting that those with HCVRF utilized more brain resources in order to support cognitive task demands. This interpretation is in line with the neural circuits hypothesis (CRUNCH), which states that greater brain activation acts as a compensatory strategy to avoid decline in cognitive performance [53].

However, our findings also showed that taking part in regular physical activity was especially beneficial for the HCVRF group, who showed lower cortical activation during SC and DT at follow-up visits, whilst improving at the cognitive tasks. After 12 months of participation in regular physical activity, both groups reported a significant decrement in the hemodynamic response that was concomitant with greater cognitive performance. Such a pattern was especially evident in the HCVRF group, who showed task-related cortical activation that matched that of the LCVRF group in the brain regions that were overactivated at baseline. Previous studies have suggested that with regular physical activity less cortical activation is needed to carry out cognitive tasks in healthy individuals [54] and in clinical populations [55,56]. Our findings extended this statement to individuals with high CVRFs, thus suggesting that this population may be particularly sensitive to the benefits of regular physical activity. Our results also underline that, regardless of type or intensity, regular physical activity is critical in maintaining brain and cognitive health, especially in those at greater risk of cardiovascular diseases, and in potentially slowing cognitive and brain decline in this population.

There are certain limitations of the current study that should be taken into consideration for future directions. Firstly, given the observational nature of the study, objective information of type and intensity of the physical activity sessions is not available. Although partially limiting, the available data are consistent with the behavioral and fNIRS results. Moreover, the physical activity classes provided at the EPIC center are designed by certified kinesiologists with the primary objective of preventing and reducing risk factors associated with cardiovascular diseases. Therefore, our participants received structured programs of physical activity. Regarding the dual-task paradigm utilized in the current study, it should be mentioned that during SC, participants were asked to complete the 2-back whilst standing. As cognitive resources involved in postural control increase in older adulthood [57], the act of standing brings a greater involvement of frontal cortical regions than in younger adults [58], as suggested by our findings of premotor and motor activation during SC. Moreover, although the ABBA design was selected to control for fatigue effects, the sequence of tasks may have had an effect on cognitive performance. Future studies should consider sitting positions in order to emphasize the difference in brain activation between single and dual tasks, and may consider alternative experimental designs. Given the small size of our sample, future investigations may consider replicating our results in bigger samples and by recruiting individuals with a greater range of CVRFs (e.g., obesity, sedentariness). With larger samples, statistically significant group differences in demographic variables (e.g., level of education, PASE) may also be detected. Finally, in our study the HbR responses failed to show significant differences between groups or time points. This is in line with the notion in fNIRS research that HbR responses may lack statistical power because they are less pronounced compared to HbO responses [59,60,61]. HbO changes are indeed believed to better reflect neurovascular coupling as they are more sensitive to task-related changes in cerebral oxygenation due to the higher changes in amplitude [59]. The absence of significant results for HbR responses may also be partially attributable to the small sample size.

Although physical activity has been recognized as one of the most cost-effective methods to prevent cardiovascular diseases [62], to the best of our knowledge, this was the first study to show its benefits on cognition and brain health in individuals with CVRFs. The results of this study suggest that participation in regular physical activity increases the efficiency of task-related hemodynamic responses and improves performance on tasks of executive functions. Future studies should further investigate physical activity as a promising method to alter the trajectory of cognitive and brain decline in older adults with high CVRFs.

## Figures and Tables

**Table 1:** Demographic, clinical information, and training characteristics.

Variables	LCVRF (14)	HCVRF (9)
Female n (%)	10 (71.4%)	5 (55.6%)
Age (years)	66.86 ± 5.63	69.78 ± 5.89
Education	16.64 ± 4.03	13.78 ± 4.18
MMSE	28.57 ± 1.16	28.11 ± 0.93
GDS	3.71 ± 3.15	6.00 ± 4.24
PASE	127.43 ± 63.90	105.64 ± 57.59
Smoking n (%)	1 (7.10%)	2 (22.20%)
Blood Parameters:		
Resting SBP (mmHg)	127.54 ± 13.87	135.11 ± 15.14
Resting DBP (mmHg)	74.92 ± 6.61	79.89 ± 9.09
Total cholesterol (mmol/L)	4.33 ± 1.25	4.04 ± 0.95
LDL-cholesterol (mmol/L)	2.30 ± 1.03	2.23 ± 0.66
HDL-cholesterol (mmol/L)	1.47 ± 0.47	1.27 ± 0.31
Medications	0.43 ± 0.94	2.67 ± 1.32
Characteristics of the 1-year physical activity:		
Frequency (n° visits/week)	1.82 ± 0.84	1.73 ± 0.92
Duration (min/week)	221.38 ± 138.18	161.33 ± 82.53
Intensity (Borg’s scale)	4.47 ± 2.21	4.00 ± 1.68

*Note.* Results are mean ± SD. MMSE = mini-mental state examination; Geriatric Depression Scale; PASE = physical activity scale for the elderly; SBP = systolic blood pressure; DPB = diastolic blood pressure; LDL = low-density lipoprotein; HDL = high=density lipoprotein.

**Table 2:** Means and standard errors of HbO responses evoked by the experimental conditions.

Experimental Condition	T0		T6		T12	
	LCVRF	HCVRF	LCVRF	HCVRF	LCVRF	HCVRF
*Single Cognitive:*						
PM	0.332 ± 0.271	**0.627 ± 0.478**	0.210 ± 0.228	0.321 ± 0.347	0.300 ± 0.356	**0.162 ± 0.202**
M	0.087 ± 0.115	0.151 ± 0.167	0.008 ± 0.082	0.070 ± 0.112	0.041 ± 0.109	0.006 ± 0.083
PFrm	0.069 ± 0.235	0.241 ± 0.248	0.052 ± 0.218	0.233 ± 0.186	0.198 ± 0.201	0.269 ± 0.241
PFrd	0.306 ± 0.557	1.176 ± 0.844	0.395 ± 0.382	0.632 ± 0.469	0.368 ± 0.415	0.691 ± 0.892
PFcd	**0.270 ± 0.474**	**0.840 ± 0.541**	0.305 ± 0.396	0.488 ± 0.374	0.440 ± 0.443	**0.229 ± 0.241**
*Single Walking:*						
PM	0.116 ± 0.492	0.331 ± 0.553	−0.021 ± 0.311	0.117 ± 0.360	0.158 ± 0.415	0.065 ± 0.235
Right M	0.054 ± 0.179	0.052 ± 0.198	0.001 ± 0.069	0.022 ± 0.146	0.0133 ± 0.121	0.055 ± 0.102
Right PFcd	0.092 ± 0.712	0.465 ± 0.771	−0.330 ± 0.697	0.055 ± 0.759	-0.024 ± 0.628	0.052 ± 0.264
*Dual Task:*						
PM	**0.703 ± 0.684**	**1.193 ± 0.821**	**0.225 ± 0.413**	**0.439 ± 0.493**	**0.555 ± 0.621**	**0.477 ± 0.557**
M	0.126 ± 0.173	0.253 ± 0.262	0.021 ± 0.107	0.078 ± 0.148	0.097 ± 0.201	0.110 ± 0.228
PFrm	0.154 ± 0.488	0.481 ± 0.472	−0.082 ± 0.468	0.321 ± 0.622	−0.054 ± 0.398	0.131 ± 0.276
PFrd	**0.864 ± 0.962**	**1.899 ± 1.410**	**0.342 ± 0.562**	**0.760 ± 0.897**	**0.555 ± 0.760**	**1.092 ± 0.943**
PFcd	0.754 ± 0.970	1.341 ± 1.036	0.355 ± 0.732	0.396 ± 0.796	0.671 ± 0.862	0.438 ± 0.823

*Note.* T0 = Baseline; T6 = 6-month follow-up; T12 = 12-month follow-up; LCVRF = low-risk cardiovascular risk factors; HCVRF = high-risk cardiovascular risk factors. Values shown in Δμmol/L. Highlighted difference between groups *p* < 0.05.

## Data Availability

Data, analytic methods, and study materials can be made available upon request.
